# Multi-Sensor Data Fusion with a Reconfigurable Module and Its Application to Unmanned Storage Boxes

**DOI:** 10.3390/s22145388

**Published:** 2022-07-19

**Authors:** Sung-Kyu Lee, Seung-Hyun Hong, Won-Ho Jun, Youn-Sik Hong

**Affiliations:** 1 Department of Computer Science and Engineering, Incheon National University, Incheon 22012, Korea; sungkyulh@naver.com (S.-K.L.); whi7ehyun@gmail.com (S.-H.H.); junwh_iot@naver.com (W.-H.J.); 2 Advanced Software Research Center, Incheon National University, Incheon 22012, Korea

**Keywords:** an unmanned storage box, multi-sensor fusion, data refinement, reconfigurable module, threshold

## Abstract

We present a multi-sensor data fusion model based on a reconfigurable module (RM) with three fusion layers. In the data layer, raw data are refined with respect to the sensor characteristics and then converted into logical values. In the feature layer, a fusion tree is configured, and the values of the intermediate nodes are calculated by applying predefined logical operations, which are adjustable. In the decision layer, a final decision is made by computing the value of the root according to predetermined equations. In this way, with given threshold values or sensor characteristics for data refinement and logic expressions for feature extraction and decision making, we reconstruct an RM that performs multi-sensor fusion and is adaptable for a dedicated application. We attempted to verify its feasibility by applying the proposed RM to an actual application. Considering the spread of the COVID-19 pandemic, an unmanned storage box was selected as our application target. Four types of sensors were used to determine the state of the door and the status of the existence of an item inside it. We implemented a prototype system that monitored the unmanned storage boxes by configuring the RM according to the proposed method. It was confirmed that a system built with only low-cost sensors can identify the states more reliably through multi-sensor data fusion.

## 1. Introduction

Sensors can continuously observe external conditions. Internal and external changes related to system operation can be tracked in real time through integrated analysis of the sensed data. Therefore, in order to make an existing system an intelligent system, it is necessary to deploy various types of sensors and effectively combine them. One of the advantages of multi-sensor data fusion is redundancy. While the same type of sensors may have different fidelities, the measured values of multiple sensors can be combined to improve accuracy and reliability. In other words, combining several low-cost and low-performance sensors may be better than one high-cost and high-performance sensor. A combination of heterogeneous multi-sensors can collect various data that are difficult to collect with only a single type of multi-sensor. In summary, an intelligent system built with a combination of several low-cost and low-performance sensors has the advantage of having a similar performance and lower cost compared to those built with high-cost and high-performance sensors.

The first step in building an intelligent system is sensor registration. This is the process of determining what kind of sensors are needed and where to place them. This registration process associates the problem to be processed using the intelligent system and the data to be sensed by the sensors. That is, it is necessary to consider whether the raw data to be sensed by each sensor will be used to extract which features.

Before sensor fusion, we need to match the raw data sensed from the sensors. That is, it is necessary to check whether the raw data are acquired under the same conditions such as the same time and the same location. A multi-sensor fusion of the same type can have a mutual effect if the value of one sensor is different from that of another under the same condition, and there may be mutual influence. In the case of heterogeneous sensor fusion, there is the problem of how to combine different information. However, in such a fusion, one sensor may directly affect the operation of another sensor. For example, in autonomous robots, vision sensors will affect the determination of the moving direction of the robot. We call this method of processing sensed data guidance (guiding) or hint (cueing).

**Figure 1:**
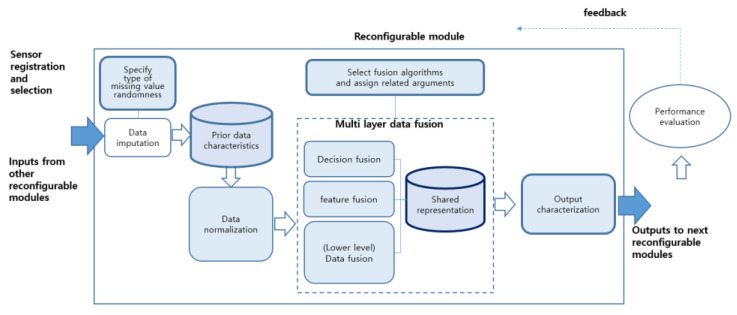
A reconfigurable module with layered architecture.

When building an entire system, it is necessary to configure a multi-sensor fusion system for the application purpose. It is easy to expand when designed in units of modules. Each module operates independently. The modular design can increase in flexibility while reducing complexity in the system integration. A layered architecture design is also needed. Sequential processing and parallel processing are possible for each layer. Although the internal representations of each layer are different from each other, it is possible to exchange information between neighboring layers. Adaptability means that a new binding model can be readily applicable to the fusion process. In this paper, we intended to implement a reconfigurable module (RM) that can be utilized for a general purpose, as shown in Figure 1, in accordance with the above-mentioned implementation purpose.

Henderson and Shilcrat [1] proposed a logic sensor model, and applied this concept to robot arm control. The existing model proposed an integrated single bonding method, but in this study, it was subdivided into a three-layer binding layer (Figure 1). In the proposed model, the task of examining outliers in individual sensor measurements was conducted in the data fusion layer, which was the lowest layer. In the upper fusion layer, we focused only on improving the accuracy of the decision making. In this paper, the term reconfigurable module (RM) is used to clearly define the meaning of the aforementioned logic sensor.

Data combination in an RM processes the raw data of the data layer into decision information of the decision layer through layer-specific fusion steps (Figure 1). The logic sensor accepts the measured value and data format of the sensor as input regardless of the type of physical sensor. It can process data regardless of the type or location of the physical sensor. Inside the logic sensor, the fusion method can be freely changed, enabling reconfiguration.

An RM’s input is either raw data or output transferred from another RM. To deal with data inconsistency, an appropriate data imputation algorithm is applied according to the random nature of the missing values in the raw data. The parameters required for setting the internal structure of the RM include data normalization, data fusion architecture, a fusion algorithm, and the internal representation and output characteristics of each fusion layer. RM reconfiguration means to create a new RM instance by setting these parameters.

An internal representation generated in the process of each fusion layer is recorded in a local database. The representation generated in the previous layer is used as input into the next layer. Notice that its format continues to change whenever layer-by-layer fusion proceeds. The state of an RM instance consists of processed data and an internal representation for each layer.

In this paper, we attempted to verify the feasibility by applying the proposed RM to an actual application. Considering the recent COVID-19 pandemic situation, we chose an unmanned storage box as our application target. Four different types of sensors were attached to it to determine the door’s status or the presence or absence of an item. In the data layer, raw data sensed by each sensor were refined based on threshold values and then converted into their corresponding logical values. In the feature layer, a tree suitable for data fusion was configured, and the logical values of the intermediate nodes were calculated by applying predefined logical operations. In the decision layer, a final decision was made by computing the logical value of the root node according to predetermined logical equations.

While integrating and fusing data from multiple sensors using the RM, data representation at each layer is important. This is because not only are the processed results through sensor fusion of each layer reflected in the data representation, but they must have data association with the adjacent upper layer to reflect them to the upper layer. That is, the reconfigurable fusion model is possible only when it has the form x→f(x)→g(f(x)), where x is the representation at the data layer. In other words, each layer behaves independently of the others, but the behavior of a higher layer depends on the representation of a lower layer through internal representation sharing. It is also possible to change only the fusion algorithm of the upper layer without changing the result of the lower layer. Therefore, the goal of the RM model is to provide a search space to find the best one by testing various fusion methods in each layer.

To verify this proposed approach, we will apply it to the unmanned storage box. This is because it can effectively limit the research scope, as well as being suitable for modeling the association between data representations of the layers.

The paper is organized as follows: Section 2 describes related works on multi-sensor data fusion. In Section 3, we describe sensor registration and its deployment and the multi-sensor data fusion process for the implementation of a smart unmanned storage box. Section 4 describes the fusion process applied to each layer. Section 5 gives the concluding remarks.

## 2. Related Works

The multi-sensor data fusion model [2,3,4,5,6] varies depending on the sensor type and application purpose. The fusion process model is a framework for combining data. Early related studies presented the results from application points of view. Since then, research results from theoretical points of view have been presented, which reflect data uncertainty in the model or pursue a model that can predict combined performance. In the JDL four-level model for military use [2], it was divided into level 1: object assessment, level 2: situation assessment, level 3: impact assessment, and level 4: process refinement. However, the most common data fusion model is the one proposed by Luo and Kay [3]. Dasarathy [4] subdivided it into five levels based on data input/output rather than data processing from a software engineering perspective. Goodman [5] attempted to reflect uncertainty in the decision-making process using random sets. Kokar [6] proposed an abstract framework that can model Luo and Kay’s four-level data fusion at once. The data fusion model [7] suitable for an ad hoc environment consisting of mobile devices or an environment in which various types of small heterogeneous sensors are deployed has also been proposed.

There was a case where a distributed matching model [8] was applied to create a tree structure for vertical combination of the data layer and the feature layer, and to score each path through a tree search. There was another case where a tree search was applied to the vertical combination of the feature layer and the decision layer. The leaf of the tree contained features extracted during the fusion process. There was also a case of applying a parameter-based cluster analysis method [9] that evaluated the similarity between decision-making results and the feature vectors stored in the leaf.

In a study related to multi-sensor fusion application, Rahman et al. [10] used a space-geometric approach to detect indoor objects by combining data from an infrared (IR) camera sensor and an acceleration sensor of a mobile device (cell phone) instead of an RFID-based method. Personalized intelligent systems are also using location-based information in more depth. Kalasapur and Kunjithapatham [11] presented a framework for personalized, context-aware applications that can dynamically fuse physical data such as from accelerometers and GIS and social network service data.

In addition, Zhao et al. [12] proposed a collaborative sensing map framework through an opportunistic network. This is because mobile devices can configure data fusion models more effectively if they can recognize their surroundings through periodic/non-periodic communication with their neighboring nodes.

Depending on the degree of randomness of the occurrence of missing values in the data imputation process, it can be classified into three categories: completely random, partially random, and non-random. According to these data characteristics, clustering, a proximity matrix using iterative partitioning [13], and FCMimpute [14] have been proposed.

Park et al. [15] developed an unmanned home-delivery box system with a weight sensor and a shock sensor. It provides a warning alarm when an external shock is detected and sends a text message to the recipient’s smartphone.

The method of extracting features from raw data can be divided into threshold value and window sampling depending on the application purpose. In multi-modal learning [16], window sampling is applied to divide stream data into segments.

A typical application of using threshold value to extract features from raw data is gait partitioning [17]. In particular, in the study of gait analysis using foot pressure insoles [18], two algorithms were proposed for phase discrimination. The first algorithm, described as force detection, discriminated between the stance phase and the swing phase, imposing a weighted threshold value obtained from the evaluation of the maximum and minimum of the force acquired during the entire cycle. The stance phase occurred when the sum of the outputs of all pressure sensors was above the threshold value. The second algorithm, described as area detection, was based on the information of which insole area was loaded. In other words, it can be seen that the features extracted from the raw data influence the final decisions. Suppose that a single pressure sensor is changed to an array of pressure sensors during the sensor deployment in a prototype system to be built. In the data layer, we will still find the sensors that measure pressure above the threshold value. However, the feature layer will extract the pressure distribution as a new feature, instead of whether there is pressure or not. In the decision layer, we will make decisions based on the area in which the pressure has occurred.

Another comparative related work is multi-modal learning [16]. This model consists of two layers. The lower layer extracts a feature vector that is an abstract representation of single modal data through learning. The upper layer learns in the direction of efficiently combining the feature vectors of each modal. It is known that the hierarchical fusion method performs better than the one that combines modal data as they are [19]. Multi-modal learning is mostly applied to information fusion [20].

In fact, the work [21] applying the multi-modal learning to the analysis of daily activity patterns can be directly compared with our proposed approach. The authors attempted to combine the data acquired from the sensors (accelerometer, blood flow sensor, electrical conductivity sensor) of a smart watch with the video data acquired from a glass-type eye tracker. They proposed a method of combining sensor data with different weights depending on the situation by placing a gate module that determines the weight of each sensor data. In summary, they proposed a method to reduce computational complexity and reduce the influence of unrelated or noisy signals through selective gate module coupling.

For our proposed approach, the results of data fusion at the feature layer can be sequentially applied to the decision layer by separating the feature layer and the decision layer through tree-based modeling. The feature layer can also provide additional information needed for decision making. During the data fusion process, some sensors are excluded in [6], but none of the sensors are excluded in the proposed approach. This is because the purpose of applying data fusion in the proposed method is to compensate for the shortcomings while maintaining the unique characteristics of the sensor data.

## 3. An Unmanned Smart Storage Box System

In Korea, as the number of single-person households continues to increase, an unmanned storage box has been recognized as a tool for receiving goods in a non-contact manner. In particular, for the case of single-female households, they are being used as a means of safely receiving ordered items without face-to-face contact with outsiders. With the prolonged COVID-19 pandemic, the demand for them has also risen along with the increased demand for parcel delivery services. Thus, it was chosen as our application target to verify the feasibility of the proposed RM.

A study of the conventional unmanned parcel delivery locker [22] focused on the opening and closing function. One can open or close its door by scanning the QR code on a recipient’s mobile phone or entering the verification digits. We attempted to implement a smart storage box by attaching specific sensors to this locker. We call it smart storage for short.

**Figure 2:**
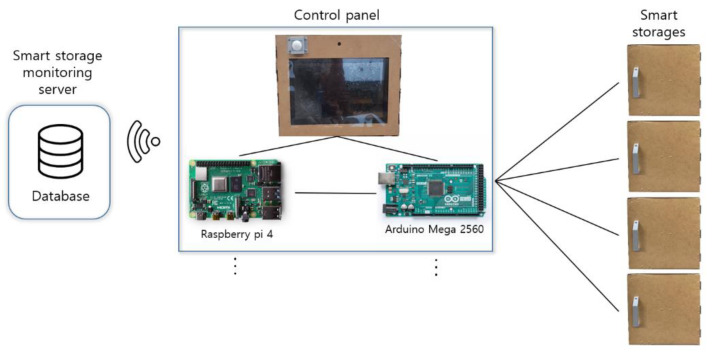
An overview of the prototype smart storage system.

The structure of the smart storage is shown in Figure 2. An Arduino Mega board (Model 2560) was used for sensor control. It receives raw data from up to 16 individual sensors. Thus, it can manage four smart storages at the same time. We used a Raspberry Pi 4 (Model B) board for processing and storing the raw data collected by the sensors. It was connected to the Arduino board by serial communication (USB 2.0). It can manage up to 16 Arduino boards. That is, it can manage 64 smart storages at the same time. Raw data temporarily stored in it were transmitted to the smart storage monitoring server via wireless LAN (IEEE 802.11a/c) and then saved in the database.

**Figure 3:**
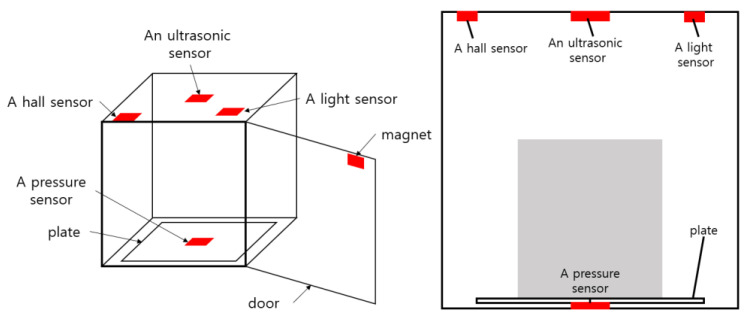
The deployment of the four sensors in the smart storage.

For implementation of the smart storage, four types of sensors were used: an ultrasonic sensor, a Hall effect sensor (abbreviated as Hall sensor), a light sensor, and a pressure sensor. Each sensor operated independently, but the status of the smart storage would be more accurately discriminated through multi-sensor data fusion. The functions and specifications of the four sensors used in the smart storage are summarized in Table 1. Considering the function of each sensor, they were deployed as shown in Figure 3.

An ultrasonic sensor was attached to the ceiling of the smart storage. It was used for determining the presence or absence of an item. As it will detect the presence of an item through collaboration with a pressure sensor, there was no need to use a high-end sensor. This is one of the advantages of multi-sensor data fusion. In this study, the HC-SR04 [23] with a maximum measurement range of four meters was used.

When a magnetic field is applied to a conductor through which a current flows, the Hall effect occurs in which voltage is generated in a direction perpendicular to the current and magnetic field. The voltage is proportional to both the current intensity and the magnetic field strength. When the current is constant, the output voltage generated is proportional to the magnetic field strength. This Hall effect allows us to know the direction and the strength of the magnetic field. We used a Hall sensor and a magnet to determine whether the door was open or closed. As shown in Figure 3, the magnet was attached to the upper left of the door, and the Hall sensor was installed on the upper left of the smart storage. That is, when the door was closed, the Hall sensor and the magnet were arranged so that they would contact each other at a close distance. The model TS0215 [24] was used as the Hall sensor.

A light sensor (SEN030101) (also called a photoresistor) [25] detected the ambient brightness. It was characterized by changes in conductivity when it received light, which is photoenergy. It was used together with the Hall sensor to determine the status of the door. It was attached to the ceiling near the door as shown in Figure 3. Since it detects the brightness inside the storage, there was no need to restrict where it was installed. In addition, the collaboration of the light sensor and the Hall sensor allowed us to check whether the door was fully closed.

When pressure is applied, a pressure sensor returns a resistance value corresponding to it. If there is no pressure, its value is infinite. Thus, it was used to check whether an item was placed in the smart storage. Eventually, it determined the presence of an item through collaboration with the ultrasonic sensor. The force-sensing resistor (FSR-406) [26], which can measure from a minimum of 0.1 N to a maximum of 100 N, was used as a pressure sensor. It was installed in the center of the floor of the smart storage. Due to its small size (38 × 38 mm), a special plate was manufactured, and it was installed on the bottom of this plate as shown in Figure 3.

Multiple FSR sensors can be arranged in an array structure to detect changes in pressure regardless of where an object is placed. However, in this study, only one FSR sensor was used to determine out how its measured value varied depending on the location of an item and the vibration generated while it was placed. We considered the case in which an item was placed away from the position where it was installed.

Notice that a different kind of sensor with a similar function can replace the existing sensor. For example, for the prototype system, an ultrasonic sensor will be used to measure the distance to an item, but it can be replaced by an infrared sensor that does the same thing. This is one of the objectives of our work.

While implementing the prototype system, sensors with a price of USD 10 or less were chosen. Thus, for this work the price standard for a low-cost sensor was set at USD 10 or less. Although the price standard of a high-cost sensor is slightly different, its price is at least 5 times higher than that of the low-cost sensors. In terms of functions, their measurement ranges are wider and they have durability such as waterproofing. The standards for low-cost sensors and high-end sensors are expected to vary depending on the application purpose.

The purpose of this study is to achieve similar effects to a single high-performance sensor through data fusion of several low-cost sensors. To this end, the low-cost sensors were used in the prototype system. However, the first thing to consider is whether these sensors can compensate each other. This is because the purpose of applying data fusion in the proposed method is to compensate for the shortcomings while maintaining the unique characteristics of sensor data.

## 4. Multi-Sensor Data Fusion with Multi-Layered Architecture

**Figure 4:**
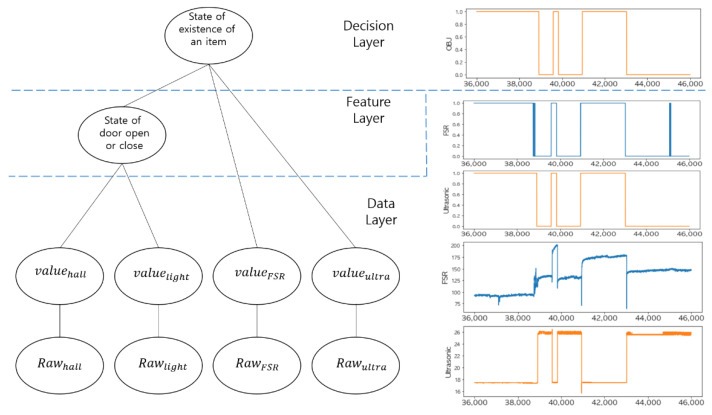
Multi-sensor data fusion with a three-layered structure.

For multi-sensor data fusion of the smart storage, we adopted a three-layer structure: a data layer, feature layer, and decision layer as shown in the left graph of Figure 4. In the data layer, it removes noises that are randomly changing and finds a significant point of data change for each sensor. This remarkable change can be inferred as a state change. A threshold, which is a reference value for judging a state change for each sensor, is obtained through multiple measurements. Based on the threshold, we can distinguish whether a state change has occurred for each sensor. That is, the threshold obtained in the data layer is applied to the measured values ($raw_{sensor}$) of each sensor and they are converted into the corresponding logical values ($val_{sensor}$), 0 and 1, as shown in the right graph of Figure 4. In the feature layer, features are extracted by combining the raw data of two different sensors that are related in terms of functionality.

The $val_{sensor}$ of each sensor in the feature layer is defined as follows.

1. $val_{FSR}$: Logical value of the FSR sensor (1: pressure detected, 0: no pressure detected).
2. $val_{ultrasonic}$: Logical value of the ultrasonic sensor (1: item detected, 0: no item detected).
3. $val_{hall}$: Logical value of the Hall sensor (1: door open, 0: door closed).
4. $val_{light}$: Logic value of the light sensor (1: light detected, 0: no light detected).

In the feature layer, the state of the smart storage door can be determined by fusing the two logical values: $val_{hall}\;$and $val_{light}$. The logical values extracted in the feature layer are transferred to the decision layer as parameters of a predefined logical expression for decision making.

In the decision layer, a final decision is made by combining the previous decision (i.e., the door status) and the features extracted from the data fusion of the two logical values: $val_{FSR}\;$and $val_{ultrasonic}$. The decision layer requires the following two decisions:

1. Has an item been placed in the smart storage?
2. Has the recipient taken the item?

An important aspect of the fusion process of the smart storage is that the current decision is affected by the previous decision. That is, if it was determined that an item was placed inside it at time t−∆t, the decision at time *t* will be made whether it was taken or not. In addition, although the event of opening the door and the event of taking out an item are independent events that occur in sequence, they can be regarded as concurrent events.

### 4.1. Sensed Data Characteristics of Each Sensor in the Data Layer

To determine the state of the smart storage, a pair consisting of an FSR sensor and an ultrasonic sensor and a pair consisting of a light sensor and a Hall sensor were combined, respectively. An FSR sensor and an ultrasonic sensor were fused together to discriminate whether an item was placed in the smart storage. Notice that when an item is placed, the distance to it measured by an ultrasonic sensor decreases. A light sensor and a Hall sensor were combined to determine the door’s state. When the door is opened, the illuminance sensed by a light sensor increases. The sensed value of the Hall sensor was 1 when the door was open and 0 when it was closed.

The purposes of multi-sensor data fusion for the above two pairs were quite different. The coupling between an FSR sensor and an ultrasonic sensor is intended to compensate for the instability of raw data. On the other hand, the coupling between a Hall sensor and a light sensor is to compensate for the functional shortcomings of each sensor.

#### 4.1.1. Sensed Data Characteristics of an FSR Sensor and an Ultrasonic Sensor

**Figure 5:**
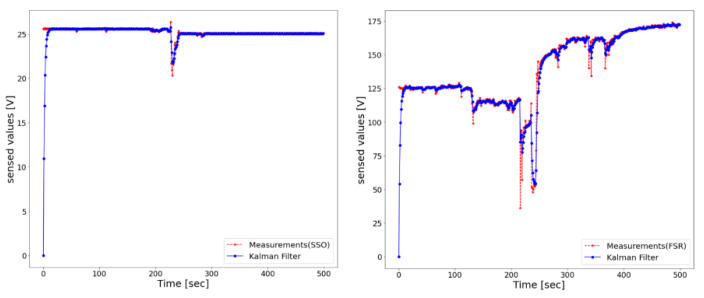
Results of applying a Kalman filter to the ultrasonic sensor (**left**) and the FSR sensor (**right**).

Figure 5 shows the results of smoothing by applying a Kalman filter [27] to the sensed values of the ultrasonic sensor and the FSR sensor. Compared to those of the ultrasonic sensor, the sensed values of the FSR sensor were relatively unstable.

**Figure 6:**
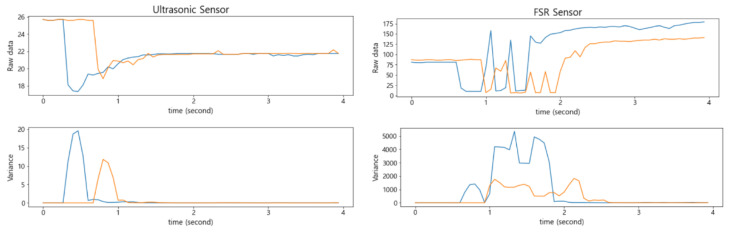
Comparison of the raw data (**top**) and the variance (**bottom**) between the ultrasonic sensor (**left**) and the FSR sensor (**right**).

At the top of Figure 6, the changes in the raw data of the FSR sensor and of the ultrasonic sensor are compared by changing the location where an item was placed in the smart storage. In addition, at the bottom of Figure 6, the variance for each of 10 measured values was obtained sequentially to show how it changed. Notice that the FSR sensor also had a larger variance than the ultrasonic sensor. In addition, it took more time to converge to a stable value as the variance decreased.

**Figure 7:**
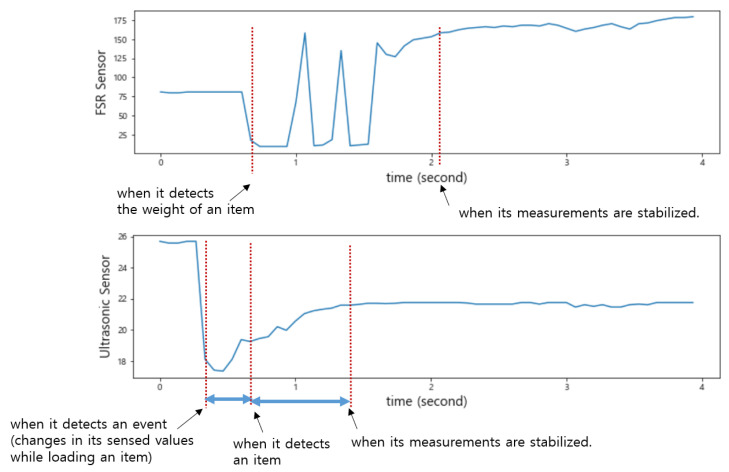
Changes in the measured values of the ultrasonic sensor and the FSR sensor when putting an item in the smart storage.

As shown in Figure 7, when an item was put into the smart storage, the ultrasonic sensor detected it or human arms first, generating noise until it was completely placed. On the other hand, when placing it, vibration occurred in the plate, resulting in a drastic change in the sensed values of the FSR sensor. We intentionally installed only one FSR sensor in the center of the plate, so its measured value changed depending on where an item was placed. In addition, the time when its sensed value converged to a stable one varied depending on the location of the item.

**Figure 8:**
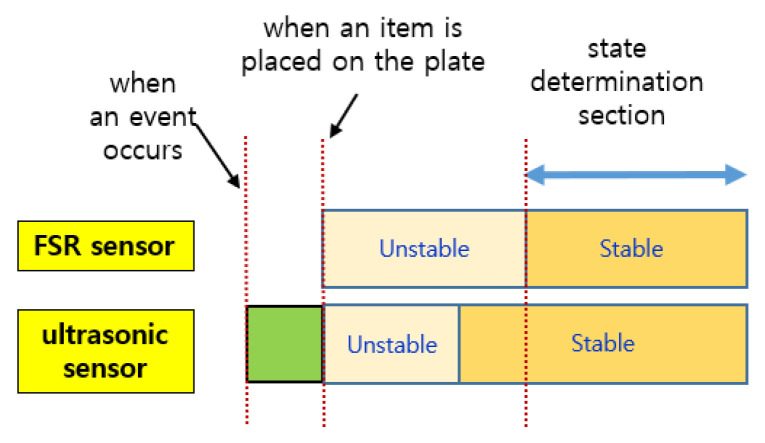
The sections for the FSR sensor and the ultrasonic sensor when putting in an item.

As shown in Figure 8, when an event occurs, the sensed values of each sensor change, but the time it takes to stabilize is shorter for the ultrasonic sensor. The FSR sensor requires a longer time to stabilize. Therefore, the reference time for determining the state of the smart storage is not the time when it detects the weight (or pressure) of an item but the time when the values of both sensors are stabilized. In addition, the one that detects an event first when putting an item in is the ultrasonic sensor. In Figure 8, the green rectangle indicates the section where the event occurred. However, the values sensed in this section are useless for state determination.

**Figure 9:**
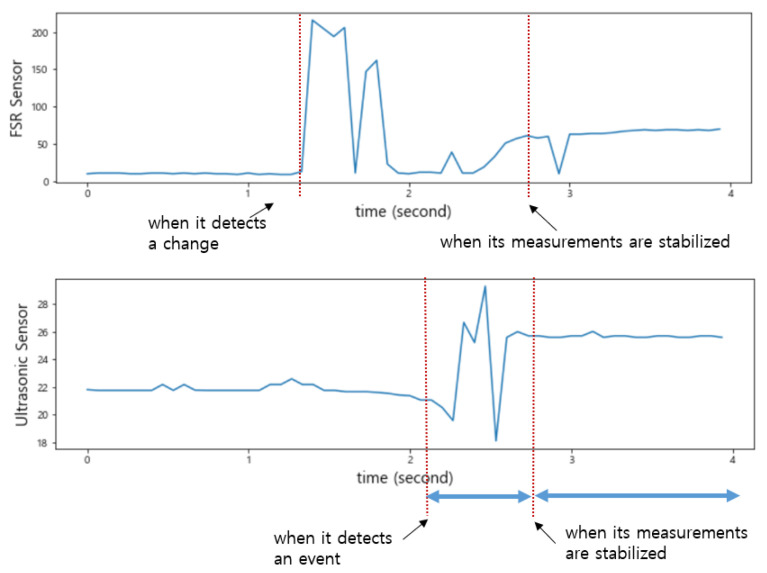
Changes in the measured values of the ultrasonic sensor and the FSR sensor when an item was taken out of the smart storage.

As shown in Figure 9, the change in the sensed values of the FSR sensor occurred first when an item was taken out of the smart storage. The time at which the sensed values of these two sensors stabilized was almost the same. Unlike when putting in an item, the vibration generated from the plate did not continue for a long time; therefore, the sensed value of the FSR sensor converged to a stable one quickly.

**Figure 10:**
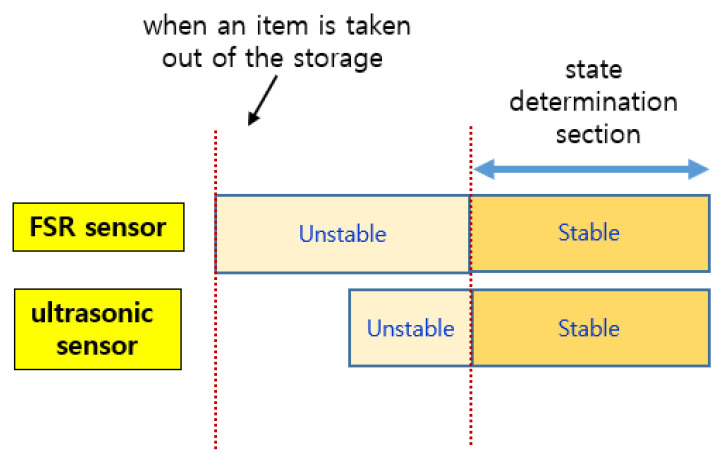
The sections for the FSR sensor and the ultrasonic sensor when an item is taken out of the smart storage.

As shown in Figure 10, when an event occurs, the FSR sensor detects it first. In addition, like the event of putting in an item, it converges to a stable value with a long delay.

#### 4.1.2. Sensed Data Characteristics of a Light Sensor and a Hall Sensor

**Figure 11:**
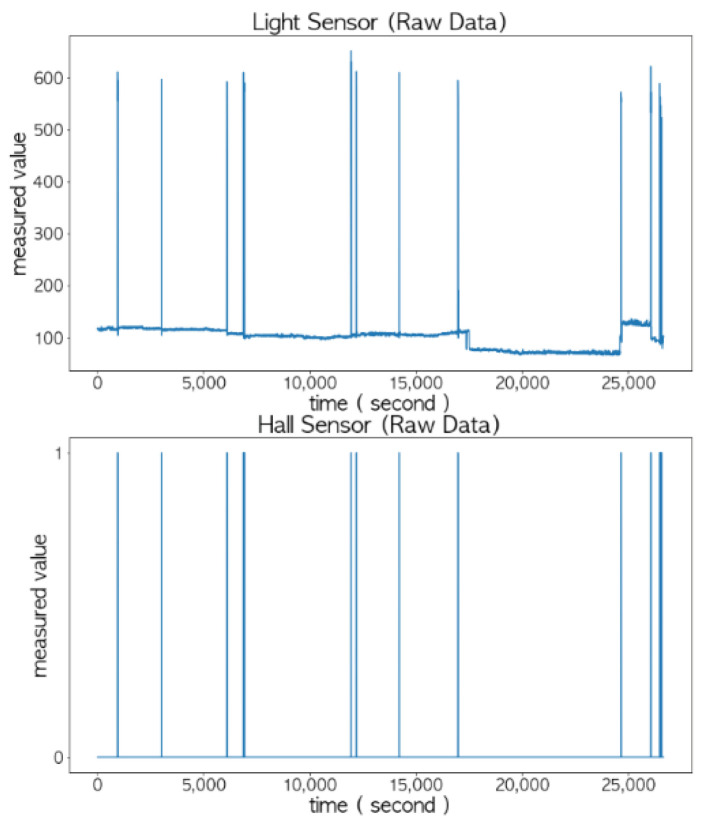
Changes in the measured values of the light sensor and the Hall sensor with respect to the opening and closing status of the door (the unit of the x-axis is in seconds).

As shown in Figure 11, the peak values of the light sensor and the Hall sensor were different, but they were combined together to accurately determine the opening and closing status of the door. The correlation between the light sensor and the door’s status and the correlation between the Hall sensor and the door’s status were 0.97 and 0.98, respectively, which are close to 1.

**Figure 12:**
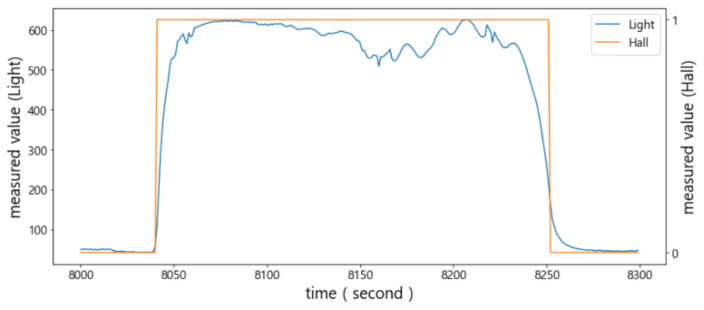
Determination of the opening and closing status of the door by combining the light sensor and the Hall sensor.

In order to analyze the sensed values of the light sensor and Hall sensor more accurately, the results of sampling 15 times per second are shown in Figure 12. Notice that typical sampling occurred once per second. The sensed values of the light sensor (i.e., orange curve) did not change rapidly and were more stable compared to the FSR sensor and the ultrasonic sensor. Thus, it is possible to determine the status of the door using only this value.

**Figure 13:**
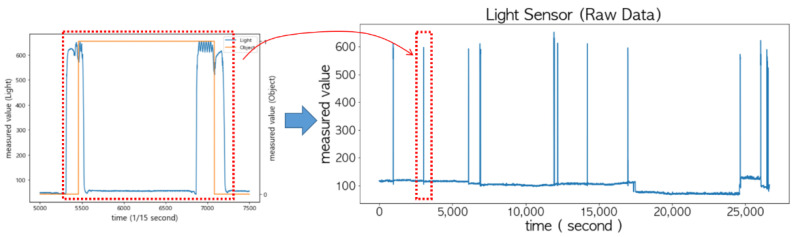
The sensed values of the light sensor: sampling 15 times per second (**left**) and sampling once per second (**right**).

When the door is opened to put an item in or take it out of the storage, a sudden change in the sensed values of the light sensor may occur, because light is blocked by the item or arms. This can easily be identified as shown in the left of Figure 13 when the sampling interval was shortened to 15 times per second. However, if the sampling interval was set to once per second, as shown on the right of Figure 13, the changes in its sensed values exactly matched the status of the door.

**Figure 14:**
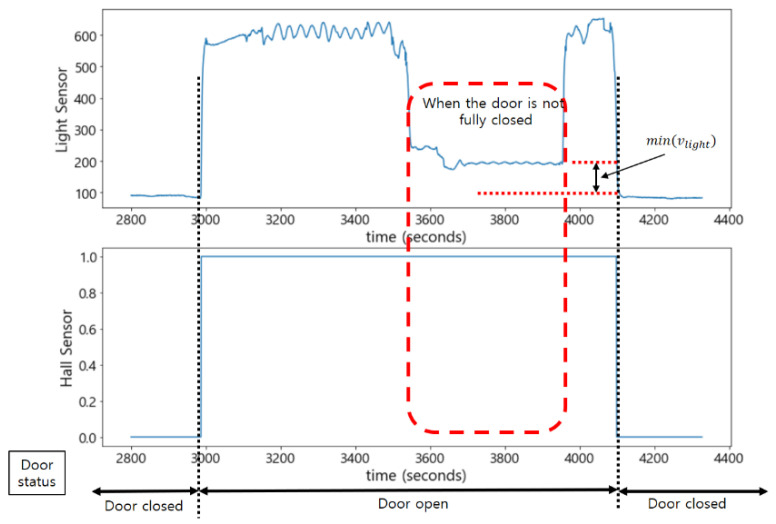
Determination of the status of the door with combination of the light sensor and the Hall sensor.

As shown in Figure 14, when the door was not fully closed, it was difficult to accurately determine the status of the door with only the light sensor. This is because its value was close to the one when the door was fully closed. On the other hand, the Hall sensor maintained the measured value 1 when the door was even slightly open; thus, it could compensate for the misjudgment by the light sensor. That is, when the measured value of the Hall sensor was 1, and the measured value of the light sensor was greater than the minimum value, $\min(v_{light})$, it was determined that the door was not fully closed.

**Figure 15:**
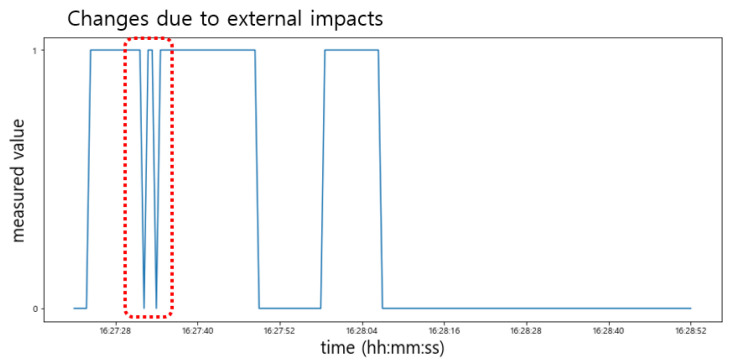
Changes in the sensed values due to the external impacts.

Conversely, as shown in Figure 15, the sensed value of the Hall sensor changed drastically (‘0′→′1′ or ′1′→′0′) due to the external impacts, especially when the door was closed. This sometimes happened when the door was opened. On the other hand, since the sensing values of the light sensor did not change in response to such an impact, it can sufficiently compensate for this phenomenon. For example, if the sensing value of the light sensor is maintained below $\min(v_{light})$, it can be determined whether the door is closed regardless of the changes in the Hall sensor.

#### 4.1.3. Multi-Sensor Data Fusion for the Smart Storage

As explained earlier, the ultrasonic sensor can sense a person’s arms in the process of placing an item on the plate; therefore, its measured value cannot be trusted until the item is fully placed. A similar case may happen when taking an item out of the smart storage. Compared to the ultrasonic sensor, the FSR sensor had a wider range of measurement values, so even minor changes could be detected. Conversely, the ultrasonic sensor had a smaller variance compared to the FSR sensor, so the time to converge to a stable value was short. Therefore, combining the FSR sensor and the ultrasonic sensor was effective for determining the presence of an item.

Typically, either the light sensor or the Hall sensor could identify the status of the door. However, the light sensor could not properly detect a state in which the door was not fully closed. In addition, the Hall sensor sometimes presented the phenomenon in which its measured values changed abruptly due to the external impacts when the door was closed. Therefore, identifying the door’s status was effective by combining the light sensor and the Hall sensor.

In some cases, multi-sensor fusion has been attempted using non-linear Kalman filters [27]. However, in the case of the smart storage, it is difficult to induce a physical relationship between the sensors to be combined. The proposed method of multi-sensor data fusion for the smart storage is to convert the raw data of each sensor into their logical values based on a threshold or operational characteristics in the data layer. Then, these logical values will be transferred to the upper layers, the feature layer and the decision layer, to obtain a reliable decision by performing predefined logical expressions. In addition, in order to perform multi-sensor data fusion using the proposed reconfigurable module, the hierarchical coupling scheme is effective.

#### 4.1.4. Analysis of Sensor Characteristics in the Data Layer Using Various Items

**Figure 16:**
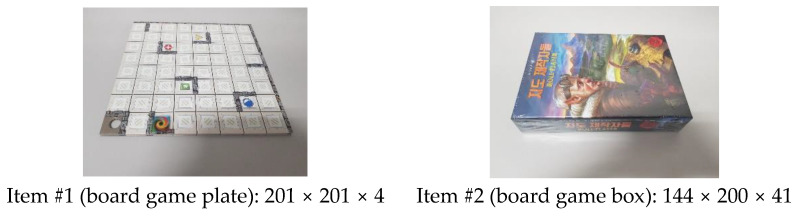
Photos and dimensions of the two items used for sensor characterization (unit: mm).

Experiments were conducted using various items to analyze the characteristics of each sensor. The light sensor and the Hall sensor were excluded from this analysis, because the door’s status was not affected by the type of item. Thus, we conducted experiments focusing on items that could compare changes in the raw data between the FSR sensor and the ultrasonic sensor. Among the items used in these experiments, the actual photos and the dimensions for two representative items are shown in Figure 16. Note that the Korean notation on the board game box means cartographer.

**Figure 17:**
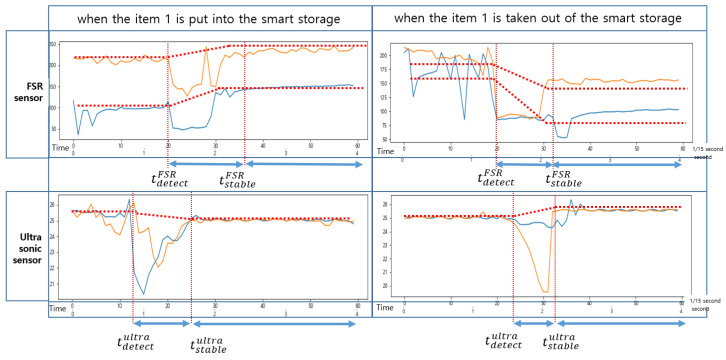
Changes in the sensed values of the FSR sensor and the ultrasonic sensor when item 1 was put in and taken out of the smart storage.

**Figure 18:**
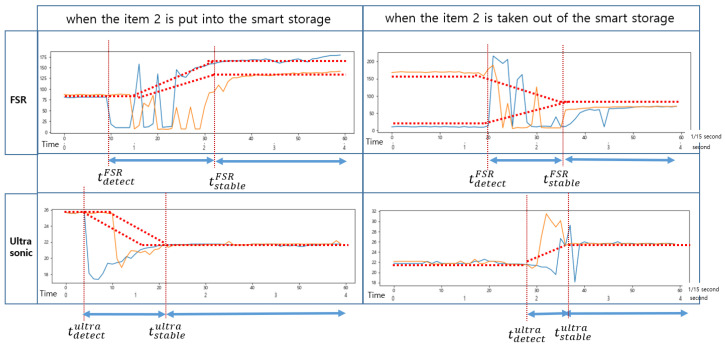
Changes in the sensed values of the FSR sensor and the ultrasonic sensor when item 2 was put in and taken out of the smart storage.

The changes in the sensed values of the FSR sensor and the ultrasonic sensor when items 1 and 2 were put in and taken out of the smart storage are shown in Figure 17 and Figure 18, respectively. Notice that $t_{detect}^{FSR}$ and $t_{stable}^{FSR}$ denote the time at which the FSR sensor detected an event and the time at which it began to converge to a stable value, respectively. Similarly, $t_{detect}^{ultra}$ and $t_{stable}^{ultra}\;$denote the time at which the ultrasonic sensor detected an event and the time at which it began to converge to a stable value, respectively. In general, the condition $t_{detect}^{ultra}<t_{detect}^{FSR}$ was satisfied in the event of putting in an item and $t_{detect}^{FSR}<t_{detect}^{ultra}$ in the event of taking out an item. In Figure 17 and Figure 18, the sampling period was 1/15 of a second, and the time in seconds is also indicated for comparison.

A basic error compensation scheme for the proposed approach is based on a sensor whose sensed value stabilized later when combining two sensors. Although this scheme is easy to implement, it is not suitable for situations where real time is emphasized due to delayed data fusion.

Another error compensation scheme is to reduce the possibility of error propagation by first combining the noisy sensor pair in the feature layer in the tree structure. This model is intended to reflect sequential fusion or concurrent fusion. In the prototype system, sequential data fusion was applied because the sequence of the events is more important than the possibility of error propagation.

### 4.2. Data Refinement at the Data Layer

Data refinement is the process of removing noise in order to extract meaningful features from raw data measured by sensors. Data refinement at the data layer is important to effectively perform multi-sensor data fusion in the upper layers. As each sensor has a different data acquisition process and sensing method, it varies from sensor to sensor. The best refinement method is to observe changes in the measurement values over a long period of time to find the threshold for refinement when an event occurs. We analyzed such changes in the measured values by intentionally generating events for each sensor. Measurements were repeated at least 20 times for each sensor, and based on this, the threshold for data refinement was determined.

#### 4.2.1. Data Refinement for the Light Sensor

The measured values of the light sensor were 70 to 130 when the door was closed. Its minimum value, $\min(v_{light}),$ was set to 130. As light entered from the outside when the door was opened, its measured value became at least 500, which was remarkably different from the one when the door was closed. Since the difference in its measured value was large depending on the open and closed state of the door, an average value of 250 was used as the threshold for determining the state change. Notice that it is determined that the door was not fully closed when the measured value of the Hall sensor was 1, and the value of the light sensor was greater than $\min(v_{light})$.

#### 4.2.2. Data Refinement for the Hall Sensor

The measured value of the Hall sensor was 1 when the door was closed and 0 when the door was open. Thus, there was no need to specify a threshold for it. Remember that when the door was closed or opened, its values sometimes changed abruptly, repeating 0 and 1, due to the external impacts. Notice that it was determined that the door was closed regardless of its changes when the values of the light sensor were maintained below $\min(v_{light})$. Similarly, it was determined that the door was open regardless of its changes when the values of the light sensor were maintained above the threshold of 250.

#### 4.2.3. Data Refinement for the Ultrasonic Sensor

An ultrasonic sensor measures the distance to an item. When there was no item, the distance that could be measured by it was the maximum, 250 mm, which was almost the same as the height of the smart storage. When an item was placed, the measured distance became shorter. The measured distance increased when an item was taken that was placed in the storage. The threshold of it was the difference between the distance measured before and after the event. In addition, depending on whether the threshold was positive or negative, it was possible to know whether an item was placed or taken. This value varied depending on the height of the item as shown in Figure 16. To determine the existence of an item based on the threshold, a delay time was required to converge to a stable value.

#### 4.2.4. Data Refinement for the Pressure Sensor

As explained in Section 3, an FSR-406 sensor measures pressure. The range of its measured value was 0 (0 N) to 1023 (100 N). Since only one FSR sensor is used to measure pressure, the measured values vary greatly depending on the where an item is placed as shown in Figure 17 and Figure 18. Compared to the ultrasonic sensor, it takes more time to converge to a stable value. However, it is effective in recognizing the occurrence of events because of its wide range of measurement. Like the ultrasonic sensor, the threshold of it is the difference between the pressure value measured before and after an item is placed. Similar to the ultrasonic sensor, a delay time is required until the measured value converges to a stable one. It depends on the characteristics of the FSR sensor,$\;\left|t_{stable}^{FSR}-t_{detect}^{FSR}\right|$. Empirically, it took about 1~1.2 s to take out an item, but about 1.4~1.6 s to put it in. In actual implementation, the longest time was applied as the delay. Notice that this delay is equally applied to sample the value of the ultrasonic sensor.

#### 4.2.5. Data Refinement Results by the Sensors

**Figure 19:**
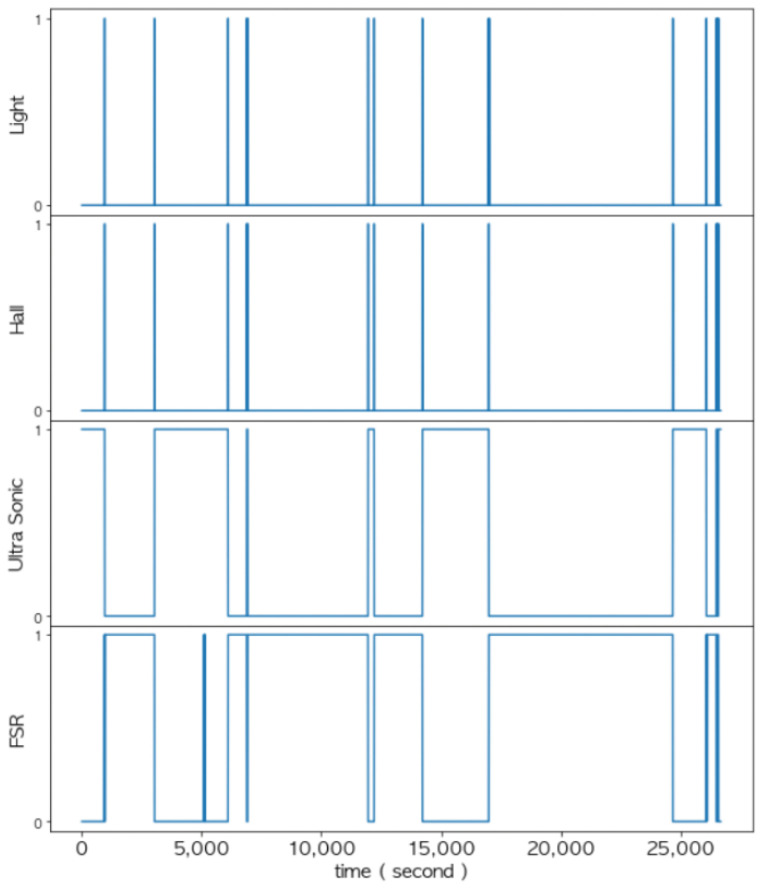
A set of graphs that refine the measurement values of each sensor into logical values.

**Figure 20:**
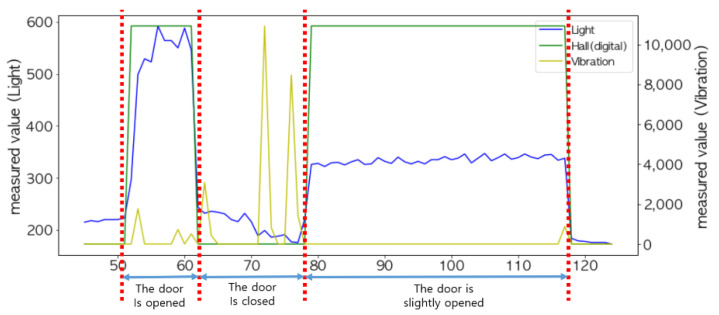
Determination of the state of the door ($state_{door}$) of the smart storage.

**Figure 21:**
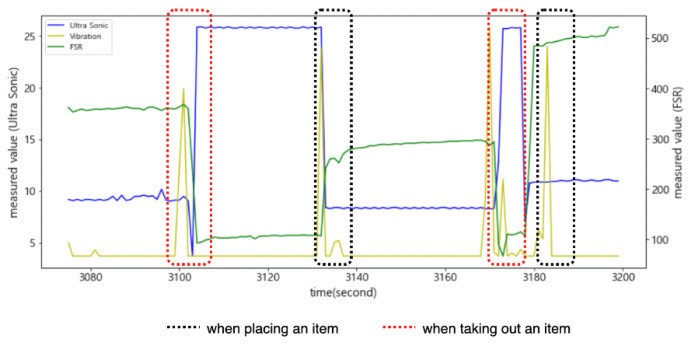
Changes in the sensed values when placing an item in and taking it out from the smart storage.

**Figure 22:**
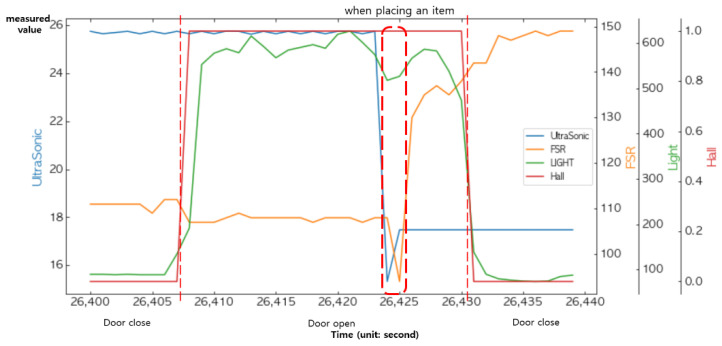
Changes in the sensor values when placing an item.

**Figure 23:**
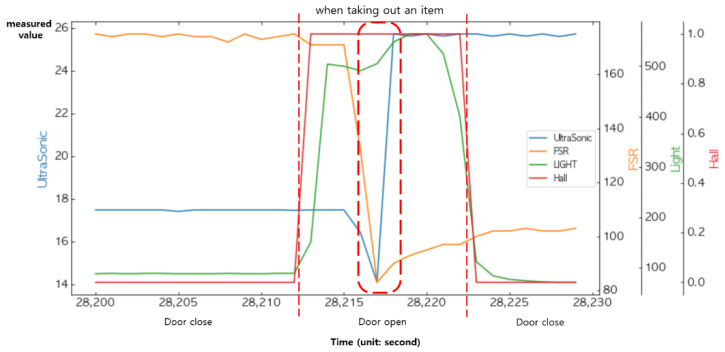
Changes in the sensed values when taking out an item.

**Figure 24:**
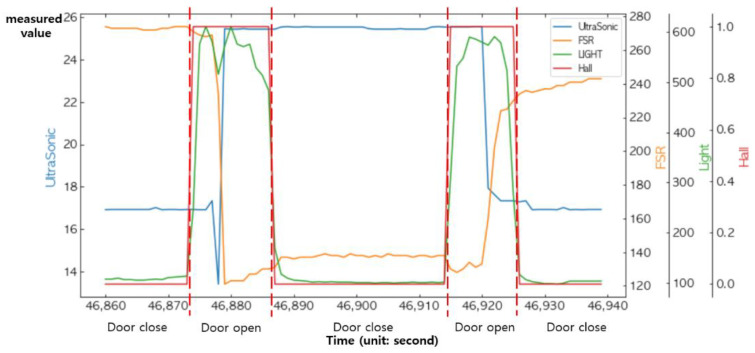
Changes in the sensed value measured by all of the sensors.

In the data layer, the measurement values of each sensor were refined based on the threshold and converted into corresponding logical values as shown in Figure 19. These logical values of each sensor were used for multi-sensor data fusion in the feature layer and decision layer. Notice from Figure 19, Figure 20, Figure 21, Figure 22, Figure 23 and Figure 24 that the sensed values of each sensor were sampled at an interval of once per second. Thus, the unit of the x-axis in the figures is seconds.

It takes about 66 ms to transmit the sensor values collected from the Arduino board to the Raspberry Pi board. It depends on the measurement time to sense values by the ultrasonic sensor. In addition, it takes about 11 ms to save the measured data from the Raspberry Pi board to the database of the monitoring server. That is, it takes a total of 77 ms to save the measured data to the database. The time it takes to retrieve the stored value from the database is about 68 ms. Finally, the time it takes to see the sensed value in the monitoring server is 145 ms, and the total latency is within 0.2 s. Notice that the data size of 24 bytes has little impact on latency.

### 4.3. Feature Extraction at Feature Layer

As described earlier, the goal of the feature layer is to determine the door status. This is because, when the door is open, it moves to the state of determining whether an item exists. In order to extract such a feature, the logical values of $val_{light}$ and $val_{hall}$ must be fused together. Through the combination of these two sensors, the accuracy of determining the door’s status can be increased.

Let $raw_{light}\left(t\right)$ and$\;threshold_{light}$ be the measured value at time *t* and the threshold of the light sensor, respectively. Similarly, $raw_{hall}\left(t\right)$ can be the measured value at time t of the Hall sensor.

1. Door open state: When $val_{hall}$ = 1 AND $val_{light}$ = 1, where it satisfies the condition $raw_{light}\left(t\right)\geq threshold_{light}$.
2. Determination of the state that the door is still open (because it is not fully closed): When $val_{hall}$ = 1 and $val_{light}\;$= 1, where it satisfies the condition $\min(v_{light})<raw_{light}\left(t\right)<threshold_{light}$.
3. Door closed state: When $val_{hall}$ = 0 AND $val_{light}$ = 0, where it satisfies the condition $raw_{light}\left(t\right)<threshold_{light}$.
4. Determination of the state that the door is still closed (even though the Hall sensor changes abruptly due to the external impacts): When $val_{light}$ = 0 AND $val_{hall}$ = 0, where it satisfies the condition $raw_{hall}\left(t+∆t_{hall}\right)$ = 0, where $∆t_{hall}$ is the delay of the Hall sensor (typically one second).

The state of the door ($state_{door}$) can be defined by the following logical AND operation of $val_{hall}$ and $val_{light}$.

(1) $$state_{door}=\mathrm{AND}\;(val_{hall},\;val_{light})$$

As shown in Figure 20, the change in the measured values between the Hall sensor and the light sensor exactly coincided with the time when the door opening and door closing event occurred. Additionally, the fusion of these two sensors could detect a state in which the door was slightly opened. Notice that a vibration sensor was additionally installed to confirm the occurrence of an event.

### 4.4. Decision Making through Logical Operations at the Decision Layer

Figure 21 shows the changes in the measured values of the FSR sensor and the ultrasonic sensor when placing an item into the smart storage or taking it from the storage. We fused these two sensors together to determine the presence or absence of an item. On the other hand, the existence of an item was an event that occurred only when the door was open; thus, $state_{door}$ must also be combined. Notice that in Figure 21, when an item was placed or taken, the measured values of the vibration sensor also changed.

When an item was placed, the distance measured by the ultrasonic sensor decreased compared to when there was no item. Based on this, in order to determine the existence of an item, the conditions of $val_{FSR}$ and $val_{ultra}$ were derived as follows:

1. The state of presence of an item after placing an item: $val_{FSR}\;$= 1 AND $val_{ultra\;}$= 1, where it satisfies the condition $raw_{FSR}\left(t+∆t_{loading}\right)-raw_{FSR}\left(t\right)>0$ AND $raw_{ultra}\left(t+t_{loading}\right)-raw_{ultra}\left(t\right)<0$, where $∆t_{loading}$ is the delay when loading to converge to a stable value (typically 1.4~1.6 s).
2. The state of no item after taking out an item: $val_{FSR}\;$= 0 AND $val_{ultra\;}$= 0, where it satisfies the condition $raw_{FSR}\left(t+∆t_{unloading}\right)-raw_{FSR}\left(t\right)<0$ AND $raw_{ultra}\left(t+∆t_{unloading}\right)-raw_{ultra}\left(t\right)>0$, where $∆t_{unloading}$ is the delay when unloading to converge to a stable value (typically 1~1.2 s). Notice that $∆t_{unloading}<∆t_{loading}$.

Figure 22 and Figure 23 show changes in all of the four sensors when an item was put into the smart storage and when it was taken out, respectively. Since the door must be opened in any situation, remarkable changes occurred in the light sensor and the Hall sensor before the event occurred.

Let us define two states as follows:

1. $state_{current}$: The state of the current decision to be determined;
2. $state_{previous}$: The state of the previous decision already determined.

Each state had a value of 1 (*there is an item*) or 0 (*there is no item*). In the decision layer, the final state $state_{current}$ was obtained through the logical operation of the three values (i.e., $state_{door}$, $val_{FSR}$ and $val_{ultra}$) and the previous decision state ($state_{previous}$), which were converted into logical values in the feature layer.

In order to place an item in the storage or take it out from the storage, the door must be open. Therefore, the condition $state_{door}\;$= 1 must be satisfied first. There is no state change when it is closed, that is, $state_{door}\;$= 0. The decision-making condition varies depending on the previous state, $state_{previous}$. Therefore, the $state_{current}$ can be determined by the following logical operations:

1. $state_{current}=(state_{current}\left(0\to 1\right)\;$**OR**$state_{current}$(1→0)) with $state_{door\;}$= 1;
2. $state_{current}\left(0\to 1\right)\;$= ($val_{FSR}$ **AND** $val_{ultra}$) with $state_{previous\;}$= 0;
3. $state_{current}$(1→0) = ($state_{current}$ **AND** ($val_{FSR}$ **OR** $val_{ultra}$)) with $state_{previous}=1.$

Note that **AND** and **OR** represents a logical AND and a logical OR operation, respectively. According to the logical value of the FSR sensor and of the ultrasonic sensor, the state of determining whether an item was present was changed. When either of them changed to the logical value of 0, the state changed to $state_{current}\left(1\to 0\right)\;$from $state_{previous}=1.$

Given the logical values of the $state_{previous}$, the $state_{door}$, the FSR sensor, and the ultrasonic sensor, the current state will be updated by the above equations. A series of events occurred in which an item was placed in the storage and taken from the storage, and the values measured by all of the sensors are shown together in a single graph in Figure 24.

## 5. Conclusions

In this paper, we proposed the use of a multi-sensor data fusion process with a three-layer structure to build a reconfigurable module (RM). Theoretically, the fused results at the feature layer can be reflected in the decision layer. That is, it is possible to build a systematic process in which the fused result at a lower layer is reflected to an adjacent upper layer. Even if individual sensors are replaced with different models, the entire fusion process can be reconstructed with only partial modifications. For an efficient reconstruction of the fusion process, the important factors (i.e., threshold values and logical equations) are parameterized. This is the main advantage of the RM model proposed in this paper.

Our fusion process was applied to an actual application, a prototype system of monitoring unmanned storage boxes, to analyze problems that arise in practical situations. A total of four heterogeneous sensors were used to implement a smart storage box. A Hall effect sensor and a light sensor were used together to determine the state of the door, and a pressure sensor (or FSR sensor) and an ultrasonic sensor were used together to determine whether an item was placed in the smart storage. In addition, in order to test the feasibility of the RM model, the feature extraction at the feature layer and the decision at the decision layer were simplified to logical expressions. To this end, the measured values of each sensor at the data layer were converted into corresponding logical values and then transferred to the feature layer. Since the Hall sensor only had values of 0 and 1, there was no need for conversion. The light sensor had a stable measured value; therefore, it was possible to convert it into a logical value based on the threshold, which was set through a series of experiments. On the other hand, the measured values of the FSR sensor and the ultrasonic sensor, which were unstable, were converted into logical values based on the difference between the value at the time of the event and the value after a predetermined delay.

In the actual implementation, the light sensor could not properly distinguish the state in which the door was not fully closed, whereas the Hall sensor was unstable due to the vibration caused by external impacts, especially when the door was closed. Compared to the ultrasonic sensor, the FSR sensor had a wider range of measurement values; thus, even minor changes could be detected. Conversely, the ultrasonic sensor had a smaller variance compared to the FSR sensor; therefore, the time to converge to a stable value was short. It was confirmed that the state could be determined more stably by compensating for the shortcomings of each sensor through multi-sensor data fusion. If the condition of the state determination is changed according to the application’s purpose, the logical expression at the feature layer and at the decision layer must also be changed accordingly. The representation of data acquired or processed at each layer changes, indicating that deriving a data correlation between layers plays an important role in the fusion process.

The RM presented in this work was configured based on logical expressions. However, if the representation of the lower layer is changed, the fusion method of the upper layer can be changed in various ways. In addition, even if the data layer has not changed, the RM can be reconfigured by changing the fusion method in the upper layer. The tree-based RM implementation proposed can be more efficient in combining heterogeneous sensors than homogeneous sensors.

The threshold-based feature extraction proposed in this paper has the advantage of simplifying features and expressing data fusion in the feature layer and decision layer with logical expressions. However, it does not reflect various characteristics of raw data in the abstraction process for high-performance sensors. In future research, we will consider a method to extract triple values by applying two distinct threshold values. In this case, a formula that reflects weights on the feature of each sensor depending on its characteristic will be effective.

We primarily used stream data. We also considered extracting segments of raw data by applying window sampling. Segment-by-segment analysis allows us to characterize variance, maximum, minimum, and means of raw data. Since the prototype system implemented in this study was a simplified model, the segment extraction focused on variance to determine the stability of the raw data. For more complex models, this segmentation technique is fully applicable.

## Figures and Tables

**Table 1:** The functions and specifications of the sensors used in the smart storage.

Sensor Type (Model)	Functions	Specification
Ultrasonic Sensor (HC-SR04)	Measures the distance of a target object by emitting ultrasonic sound waves	Ranging distance: 2~400 (cm) Size: 2.0 × 4.5 × 1.5 (cm)
Pressure Sensor (FSR-406)	Detects forces when pressure is applied	Sensing range: 0.1 to 20 (N) Size: 38.1 × 38.1 (mm)
Light Sensor (SEN030101)	Detects the ambient brightness	Sensing range: 0~1023 Size: 23 × 21 (mm)
Hall Sensor (TS0215)	Detects the strength of the magnetic field	Sensing range: logical values (0 or 1) Size: 2.0 × 2.0 (cm)

## Data Availability

All data used in this paper are dependent on the sensors used and the measurement environments. The measurement values of each sensor used in our experiments will be provided upon request by e-mail.
